# *Plasmodium falciparum* parasitaemia and clinical malaria among school children living in a high transmission setting in western Kenya

**DOI:** 10.1186/s12936-016-1176-y

**Published:** 2016-03-11

**Authors:** Stella Kepha, Birgit Nikolay, Fred Nuwaha, Charles S. Mwandawiro, Joaniter Nankabirwa, Juliet Ndibazza, Jorge Cano, Damaris Matoke-Muhia, Rachel L. Pullan, Elizabeth Allen, Katherine E. Halliday, Simon J. Brooker

**Affiliations:** School of Public Health, Makerere University College of Health Sciences, Kampala, Uganda; London School of Hygiene and Tropical Medicine, London, UK; Eastern and Southern Africa Centre of International Parasite Control, Kenya Medical Research Institute (KEMRI), Nairobi, Kenya; Department of Internal Medicine, Makerere University College of Health Sciences, Kampala, Uganda; Centre of Biotechnology Research and Development, KEMRI, Nairobi, Kenya; KEMRI-Wellcome Trust Research Programme, Nairobi, Kenya

**Keywords:** Malaria, Anaemia, School children, Kenya

## Abstract

**Background:**

Malaria among school children is increasingly receiving attention, yet the burden of malaria in this age group is poorly defined. This study presents data on malaria morbidity among school children in Bungoma county, western Kenya.

**Method:**

This study investigated the burden and risk factors of *Plasmodium falciparum* infection, clinical malaria, and anaemia among 2346 school children aged 5–15 years, who were enrolled in an individually randomized trial evaluating the effect of anthelmintic treatment on the risks of malaria. At baseline, children were assessed for anaemia and nutritional status and information on household characteristics was collected. Children were followed-up for 13 months to assess the incidence of clinical malaria by active detection, and *P. falciparum* infection and density evaluated using repeated cross-sectional surveys over 15 months.

**Results:**

On average prevalence of *P. falciparum* infection was 42 % and ranged between 32 and 48 % during the five cross-sectional surveys. *Plasmodium falciparum* prevalence was significantly higher among boys than girls. The overall incidence of clinical malaria was 0.26 episodes per person year (95 % confidence interval, 0.24–0.29) and was significantly higher among girls (0.23 versus 0.31, episodes per person years). Both infection prevalence and clinical disease varied by season. In multivariable analysis, *P. falciparum* infection was associated with being male, lower socioeconomic status and stunting. The risk of clinical malaria was associated with being female.

**Conclusion:**

These findings show that the burden of *P. falciparum* parasitaemia, clinical malaria and anaemia among school children is not insignificant, and suggest that malaria control programmes should be expanded to include this age group.

**Electronic supplementary material:**

The online version of this article (doi:10.1186/s12936-016-1176-y) contains supplementary material, which is available to authorized users.

## Background

Significant progress in malaria control has been realized in the last decade with a number of countries, including Kenya, reporting decline in malaria transmission and hospital admission [1, 2]. The progress observed is largely due to the increase in access and use of proven malaria control interventions, combined with social and economic development [3–7]. Despite this reduction, malaria remains an important public health problem worldwide with endemicity in over 100 countries in the tropics and subtropics [8, 9].

Malaria prevention typically targets the highest risk groups; pregnant women and children under five who are most affected by the severe forms of the disease. However, there is increasing evidence suggesting that school-aged children (5–15 years) bear the highest burden of asymptomatic malaria irrespective of the transmission setting with a prevalence range between 14 and 64 % [10–14], and constitute nearly half of the population at risk of malaria [8]. Moreover, due to a decline in transmission and exposure in some areas, the peak age of clinical attacks of malaria is shifting from very young (under five) to older children [3, 15].

Both asymptomatic and clinical malaria have been shown to have a negative impact on the health and cognitive development of school children [16–22]. Although, school children are included in standard malaria intervention strategies, often they have the lowest coverage of malaria preventive measures, such as bed nets use [23, 24]. While reduction of infections is observed in younger age groups that are targeted by interventions, parasite prevalence among school aged children may even increase, as observed after the national distribution of bed nets in Kenya [25, 26]. In addition, as school children are rarely treated for these asymptomatic infections, they may contribute significantly to the infectious reservoirs of malaria [27, 28].

In order to adapt malaria control strategies to changes in transmission patterns, there is urgent need for data on asymptomatic and clinical malaria among different age groups. The present study aimed to describe patterns of *Plasmodium falciparum* infection and clinical malaria among school children living in an area of high malaria transmission in western Kenya. Specifically, this study investigated the burden of *P. falciparum* infection, clinical malaria, and anaemia, as well as associated risk factors.

## Methods

### Study area and participants

The study presents secondary analysis of longitudinal data arising from an individually randomized clinical trial evaluating the impact of repeated (every 4 months) anthelmintic treatment with albendazole on clinical malaria and malaria parasitaemia among school children [29]. The trial was conducted between February 2013 and October 2014 in Bumula District, Bungoma County, western Kenya. Malaria transmission is intense and perennial, with two seasonal peaks (May–August and November–December) associated with an increase in rainfall and predominantly caused by *P. falciparum.* A school survey conducted in 2009 reported that the prevalence of *P. falciparum* among school children in western Kenya was 21.6 % and with only 19.0 % of school children sleeping under a bed net the previous night [30]. The most recent Malaria Indicator Survey conducted in 2010 reported a slightly higher proportion of children sleeping under a bed net (28.0 %) in western Kenya and a compared to national use of 20 % [26]. There was a mass net distribution campaign that was conducted in 2011, that is reported to have increased the average net ownership from 1.0 per household to 2.6 as had been earlier [31].

The trial recruited 2346 children aged 5–15 years from 23 day schools [29]. The schools were recruited purposively based on their accessibility, all children in the selected schools were invited to be part of the study. Children with signs of severe malaria, aged >15 years, or suspected sickle-cell trait were excluded from the trial. For the purpose of the trial, children were selected based on soil transmitted helminths (STH) infection status and complemented by non-infected children to reach the required sample size, so that 1505 (64 %) had detectable STH infection. There was no difference between STH infected and uninfected children in terms of demographic parameters. However, children who did not have detectable STH infection were more likely to have *P. falciparum* infection, while parasite density was not significantly different between the two groups (Additional file 1). After 15 months of follow up, there was no difference in either the incidence of clinical malaria or prevalence of *P. falciparum* between the two treatment groups [29], therefore, all the children are combined in a single analysis in the present paper.

### Baseline assessments and follow-up of children

**Fig. 1:**
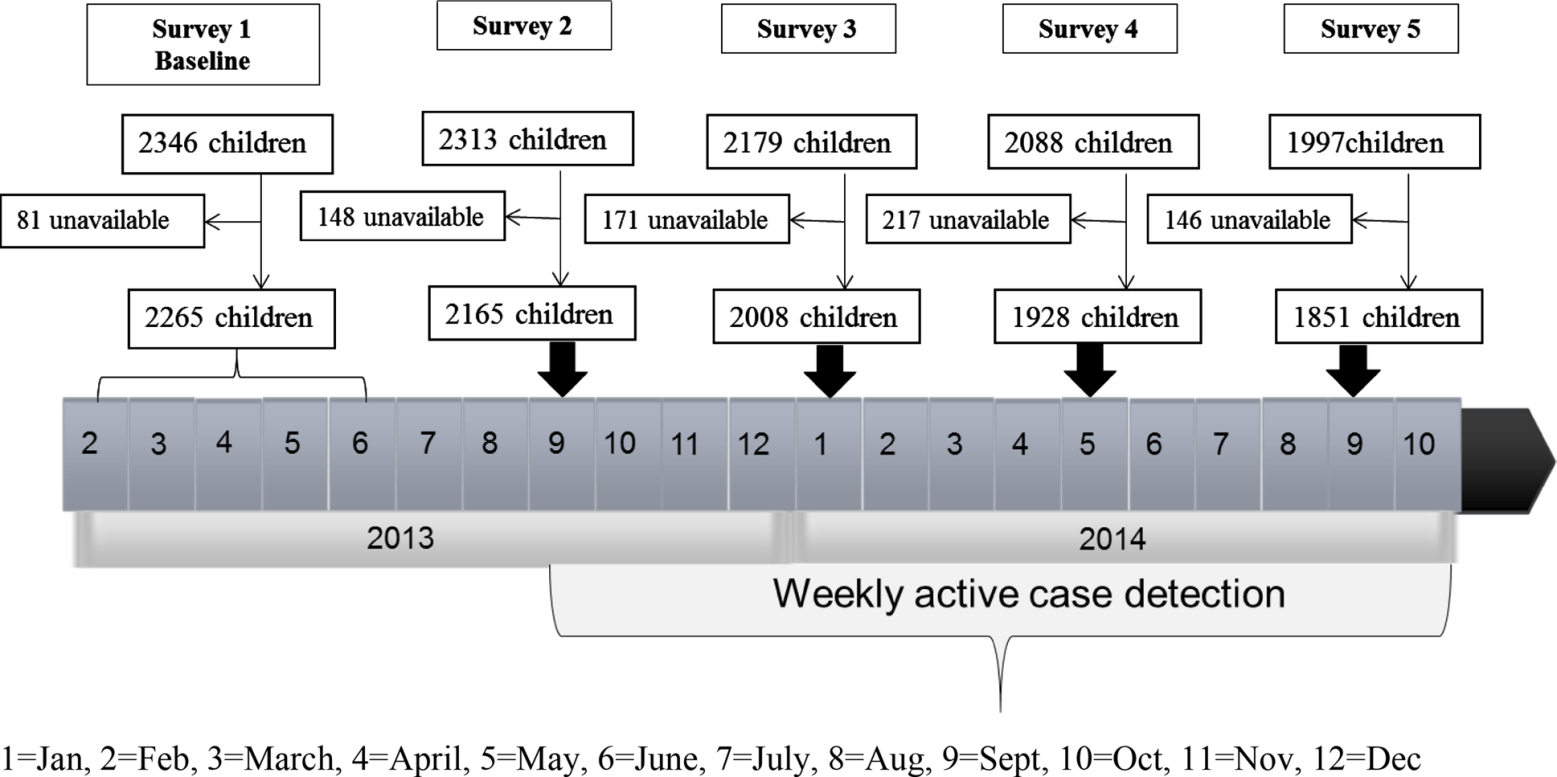
Timeline of the surveys conducted and active case detection during the 15 month follow-up period, among school children in Bumula district, western Kenya

Prior to the trial, a baseline household survey was conducted to collect information on socioeconomic characteristics, and children’s use of malaria prevention measures. During the baseline health survey, haemoglobin concentration was assessed from a finger prick blood sample using a HemoCue haemoglobin photometer (Hb 201 + , Ångelholm, Sweden), weight and height were recorded. Follow-up cross-sectional surveys at schools were conducted at 3, 7, 11, and 15 months (Fig. 1) when a finger-prick blood sample was collected from all children, irrespective of whether they had fever or not, thick and thin blood smears prepared. Active malaria case detection surveillance was conducted over 13 months of follow-up. School visits were conducted on a weekly basis, with absent children followed-up at home. Axillary temperature was measured using a digital thermometer. Children with documented fever (axillary temperature ≥37.5 °C) or who reported fever or any other signs and symptoms (headache, rigors, fevers, vomiting and chills) of malaria within the past 24 h were asked to provide a finger-prick blood sample, which was used to perform a malaria rapid diagnostic test (RDT) (Bioline Malaria Ag P.f/Pan, BD Biosciences, SanDiego, CA) and to prepare thick and thin blood smears. Children diagnosed with uncomplicated clinical malaria (fever or other malaria signs plus a positive RDT result) were treated using Coartem^®^ (20 mg artemether/120 mg lumefantrine) in accordance with national guidelines. No cases of complicated malaria were encountered.

Household and school locations of all enrolled children were mapped using a hand held GPS device (eTrex 20 Garmin Ltd., Olathe, KS, USA). Estimates of land surface temperature (LST), enhanced vegetation index (EVI), elevation and normalized difference vegetation index (NDVI) were extracted for each school after averaging the values of covariates within a 1-km catchment area around each school and household. Permanent water bodies were identified through intersecting gridded surfaces of the normalized difference water index (NDWI) for rainy and dry season. Those water areas which remain throughout the year were considered potential permanent breeding sites for mosquitoes. Straight-line distance (Euclidean distance) from schools to the nearest permanent body was calculated. A detailed description on how the data was obtained and extracted is provided in an additional file enclosed (Additional file 2).

### Microscopy and laboratory techniques

Blood smears were air dried and stained with 3 % Giemsa for 45 min. Parasite density was defined as number of *Plasmodium* parasites per μL of blood, counted against 200 leukocytes assuming a leukocyte count of 8000/μL of blood. If fewer than 10 asexual parasites were detected in the first 200 leukocytes, counting was continued against 500 leukocytes. A blood smear was considered negative when the examination of 200 high power fields failed to reveal asexual parasites. Thin smears were used for species identification. All blood slides were read by two independent microscopists with discrepancies resolved by a third microscopist.

### Ethical consideration

Written informed consent was obtained from a parent or guardian and assent was sought from children before enrollment into the study. The study was approved by the Kenya Medical Research Institute and KEMRI Ethics Review Committee (SSC no.2242), the London School of Hygiene and Tropical Medicine (LSHTM) Ethics Committee (6210), and the Makerere School of Public Health, Institutional Review Board (IRB00005876). The study was registered with the Clinical trial.gov NCT01658774.

### Definition of variables

Three malariometric outcomes were considered; (1) malaria parasitaemia defined as *P. falciparum* infection at any density irrespective of presence fever as diagnosed by expert microscopy, (2) *P. falciparum* parasite density categorized in two groups: light (1–999 parasites/μL), and heavy (≥1000 parasites/μL), and (3) clinical malaria, defined as the presence of asexual *P*. *falciparum* parasitaemia as determined by microscopy plus either an axillary temperature >37.5 °C or a reported history of fever during the preceding 24 h. Children were considered at risk from their date of entry into the study until completing follow-up at 13 months. Children with documented or reported clinical malaria or known to have received medical attention from any source other than the survey team were censored for 28 days and those children who were absent from school for ≥10 days were censored for the time of absence [29]. Anaemia was defined using age and sex corrected WHO threshold adjusted by altitude [32] with a mid-point age assumed for the self-reported age for each child as there were doubts over the correctness of the age. Patterns of *P. falciparum* infection and clinical malaria were investigated by season: wet season (May–August and November–December) and dry season (January–April and September–October).

### Individual and household-related variables

Principal component analysis was used to construct a household wealth index based on information on wall, floor, and roof construction materials, source of fuel, and education level of household head. The wealth index was then divided into two groups (termed poor and less poor) based on the median. Z-scores of height-for-age (HAZ), weight-for-age (WAZ) and body mass index for age (BMIZ) were calculated using the AnthroPlus software that uses the new 2006 WHO growth Ref. [33]. Children were classified as stunted, underweight, and thin if their HAZ, WAZ, and BMIZ, respectively, were less than −2 standard deviations (SDs) from the reference medium. Based on observed distribution, age was considered as categorical variable (5–10 years and 11–15 years). Three categories were generated to determine the consistency of bed net use; *never* (reported none use), *sometimes* (reported use at least once), and *always* (reported use at each survey) based on bed net use information collected during the five cross-sectional surveys.

### Statistical analysis

Data were analysed using Stata version 13 (Statacorp, College Station). Summary statistics were calculated for all baseline data. Proportion of children with *P. falciparum* infection and anaemia together with their 95 % confidence interval (CI) was calculated using binomial regression analysis, adjusted for clustering by school. To allow for over dispersion in the distribution of parasites, arithmetic mean parasite counts with their 95 % CIs were estimated using a negative binomial regression model taking into account school clustering. Estimates of prevalence and density of *P. falciparum* infection were calculated by sex and survey. Incidence rates were calculated as number of events divided by person years at risk using survival analysis, and were estimated for each calendar month, stratified by sex and age group. Survival analysis for clinical malaria was presented using Kaplan–Meier curves. Prevalence rate ratios (*P. falciparum* infection and anaemia) and incidence rate ratios were calculated adjusting for clustering in schools. Most of the infections were of low parasite density and preliminary analysis indicated little variation and therefore no further analysis was undertaken of this outcome.

Risk factors of *P. falciparum* infection were identified using mixed effects logistic regression modelling, where *P. falciparum* infection status was treated as repeated measures (child random intercept), with additional random intercepts to adjust for clustering of children within schools and households. As most households had a single child, the study area was divided into 191 hexagons of 1 km diameter to which households were assigned based on their geographic location (1–66 children per hexagon). Hexagon defined clusters were favored over actual household clusters due to model convergence. All univariable and multivariable models for *P. falciparum* infection included a fixed term for survey round; this adjusted for the number of times a child contributed to the analysis, even if there was missing data at a particular follow-up survey. Univariable analysis was first performed including one covariate at a time and significant variables (P ≤ 0.05, based on likelihood ratio test) were included in multivariable analysis. Parsimonious regression models were developed using a backwards variable selection approach, eliminating one variable at a time based on the highest *P* value and retaining only variables with P ≤ 0.05.

Risk factors for incidence of clinical malaria were investigated using mixed effects Poisson regression. The models included a random intercept for children, however they failed to converge when clustering at both school and household level was considered. Hence models were adjusted only for clustering at household, as this is where infection is more likely to occur. Anaemia was not included in the variable selection procedure, because of potential reverse causality. The association of *P. falciparum* infection and anaemia was however subsequently tested adjusting for variables included in the minimum model. To investigate any potential effect of previous *P. falciparum* infections on incidence of clinical malaria, the final model was further tested including the number of *P. falciparum* infections experienced by a child as a fixed term.

Factors associated with baseline anaemia prevalence were identified using mixed effects logistic regression modelling with a random intercept to adjust for clustering within schools. Model selection procedures were followed as described above.

## Results

### Baseline child characteristics

In total, 2346 children aged 5–15 years from 1712 households were included in the study. Baseline characteristics of children are presented in Table 1. At baseline, the mean age was 10.4 years [standard deviation (SD): 2.5] and 47.5 % of children were female. A total of 1853 (79 %) children reported sleeping under a bed net the previous night and the proportion did not vary between boys and girls.

**Table 1:** Baseline characteristics of study children, by sex

Characteristic^a^	Overall 2346	Boys 1232	Girls 1114	P^b^ value
Child characteristics				
Age, years, mean (SD)	10.4 (2.5)	10.6 (2.5)	10.2 (2.5)	<0.001
Age categories (years)				
5–10	50.2 (1178)	46.1 (568)	54.8 (601)	
11–15	49.8 (1168)	53.9 (664)	45.2 (504)	<0.001
Mean body temperature, mean °C (SD)	36.6 (0.6)	36.6 (0.6)	36.6 (0.6)	0.467
HAZ <−2 SD below median reference value	25.3 (593)	31.1 (383)	18.6 (210)	<0.001
WAZ <−2 SD below median reference value	3.0 (71)	3.6 (44)	2.4 (27)	0.105
BMIZ <−2 SD below median reference value	10.4 (245)	13.1 (161)	7.5 (84)	<0.001
*P. falciparum* infection	48.3 (1095)	50.8 (607)	46.7 (488)	0.015
*P. falciparum* infection density/μL, mean (95 % CI)	1886 (1452–2450)	1812 (1361–2413)	1968 (1339–2894)	0.731
Haemoglobin, g/dL, mean (SD)	12.3 (1.3)	12.3 (0.4)	12.3 (0.4)	0.142
Anaemia	38.0 (834)	40.9 (471)	34.8 (363)	0.003
Household characteristics				
>1 net per household	87.5 (1319)	85.2 (673)	90.1 (646)	0.004
Slept under a bed net previous night	78.7 (1742)	77.8 (905)	79.8 (837)	0.241
Education level of household head				
None or incomplete primary	57.4 (1255)	56.8 (653)	58.1 (602)	0.547
Above primary school	42.6 (930)	43.2 (496)	41.9 (434)	

*SD* standard deviation, *WAZ* weight-for-age z-score (underweight), *HAZ* height-for-age z-score (stunted), *BMIZ* body-mass-index-for-age z-scores, *CI* confidence interval, *μL* microlitre, *g/dL* grammes/decilitre

^a^Data are proportions % (n), unless otherwise stated

^b^ based on Wald test comparing boys versus girls

### *Plasmodium falciparum* infection

The overall prevalence of *P. falciparum* infection was 42 % and was significantly higher among boys (45 vs 39 %, P < 0.001) and among younger (5–10 years) compared to older children (11–15 years) irrespective of sex (53.9 vs 46.1 %, P < 0.001) (Table 2). The prevalence of *P. falciparum* was significantly higher during wet season compared to the dry season (42.6 vs 40.6 %, P = 0.042). The majority (68 %) of the infections were light, having parasite densities < 1000 parasites/µl of blood. Densities did not vary by sex, but varied significantly by season (P < 0.001).

**Table 2:** *Plasmodium falciparum* infection and incidence of clinical malaria, by sex among school children during the 15 months follow-up

Outcome	Events	Total tests	Prevalence	Events	Total tests	Prevalence	Prevalence ratio (95 % CI)	P value^a^
	Boys			Girls				
*Plasmodium falciparum* infection								
All events	2388	5362	0.45	1884	4853	0.39	0.87 (0.83–0.92)	<0.001
Events in dry season	2237	967	0.43	2036	770	0.38	0.87 (0.82–0.93)	<0.001
Events in wet season	3125	1421	0.45	2817	1114	0.40	0.87 (0.81–0.94)	<0.001

Outcome	Episodes	PYAR	Incidence	Episodes	PYAR	Incidence	IRR (95 % CI)	P value
	Boys			Girls				
Clinical malaria								
All episodes	277	1218.9	0.23	329	1091.8	0.31	1.33 (1.11–1.59)	0.001
Episodes in dry season	107	659.0	0.16	139	589.6	0.23	1.45 (1.12–1.88)	0.005
Episodes in wet season	170	502.2	0.30	190	560.0	0.38	1.24 (1.01–1.53)	0.036
Parasite density >2500/μL	65	1199.6	0.05	65	1068.9	0.06	1.12 (0.78–1.62)	0.225

*PYAR* person years at risk, *IRR* incidence rate ratio, *CI* confidence interval, *μL* microlitre

^a^P values based on Wald test comparing the difference between boys and girls

In univariable and multivariable analysis the odds of *P. falciparum* infection reduced with increasing age of children (P < 0.001). Additionally, *P. falciparum* infection was more common among boys, in children from households with lower socioeconomic status and who were anaemic (Table 3). Additionally, stunting was associated with increased odds of infection in multivariable analysis. However, there was no evidence for a lower risk of *P. falciparum* infection among children who slept under a bed net the previous night.

**Table 3:** Factors associated with *Plasmodium falciparum* infection, among school children in Bumula District

Variable	Crude odds ratio (95 % CI)	P value	Adjusted^a^ odds ratio (95 % CI)	P value^b^
Child characteristics				
Sex				
Male	1		1	
Female	0.78 (0.68–0.88)	<0.001	0.76 (0.68–0.87)	<0.001
Age categories (years)				
5–10	1			
11–15	0.78 (0.69–0.81)	<0.001	0.68 (0.67–0.85)	<0.001
Stunted	1.39 (0.97–1.97)	0.061	1.48 (1.04–1.41)	0.007
Underweight	1.12 (0.98–1.29)	0.095	1.22 (1.06–0.95)	0.295
Thin	0.98 (0.81–1.19)	0.848	0.96 (0.79–1.17)	0.713
Anaemia	1.17 (1.03–1.33)	0.018	1.14 (1.01–1.32)	0.030
Survey-round				
1 (Feb–June 2013)	1		1	
2 (Sept 2013)	0.71 (0.61–0.82)		0.71 (0.61–0.82)	
3 (Jan 2014)	0.46 (0.40–0.54)	0.017	0.46 (0.40–0.54)	<0.001
4 (May 2014)	0.78 (0.67–0.90)		0.78 (0.67–0.90)	
5 (Sept 2014)	0.81 (0.70–0.94)		0.80 (0.69–0.94)	
Household characteristics				
Bed net use				
Never	1		1	
Sometimes	0.99 (0.86–1.14)	0.893	0.99 (0.87–1.14)	0.990
Always	0.96 (0.81–1.12)		0.97 (0.82–1.15)	
Socio economic status				
Poor	1		1	
Not poor	0.86 (0.76–0.98)	0.026	0.85 (0.75–0.97)	0.031
LST (°C)				
16.0–20.6	1		1	
20.7–25.3	1.09 (0.85–1.39)	0.695	1.07 (0.83–1.40)	0.920
25.5–34.8	1.10 (0.80–1.52)		1.09 (0.78–1.54)	
NDVI				
0.34–0.54	1		1	
0.35–0.60	1.10 (0.96–1.26)	0.523	1.11 (0.97–1.28)	0.412
0.61–0.71	1.12 (0.96–1.31)		1.12 (0.96–1.32)	
Average distance to water bodies (km)	1.10 (0.87–1.42)	0.421	1.11 (0.88–1.42)	0.362

*LST* land surface temperature, *NDVI* normalized difference vegetation index

Stunted = HAZ < −2 SD below median reference value, thin = BMIZ < −2 SD below median reference value, underweight = WAZ < −2 SD below median reference value; wet season survey (February–June 2013, May 2014); Dry season (September 2013, January 2014, September 2014). WAZ was calculated for children 5–10 years

^a^Multivariate analysis adjusted for sex, age and socio economic status

^b^P value based on likelihood ratio test

### Incidence of clinical malaria

**Fig. 2:**
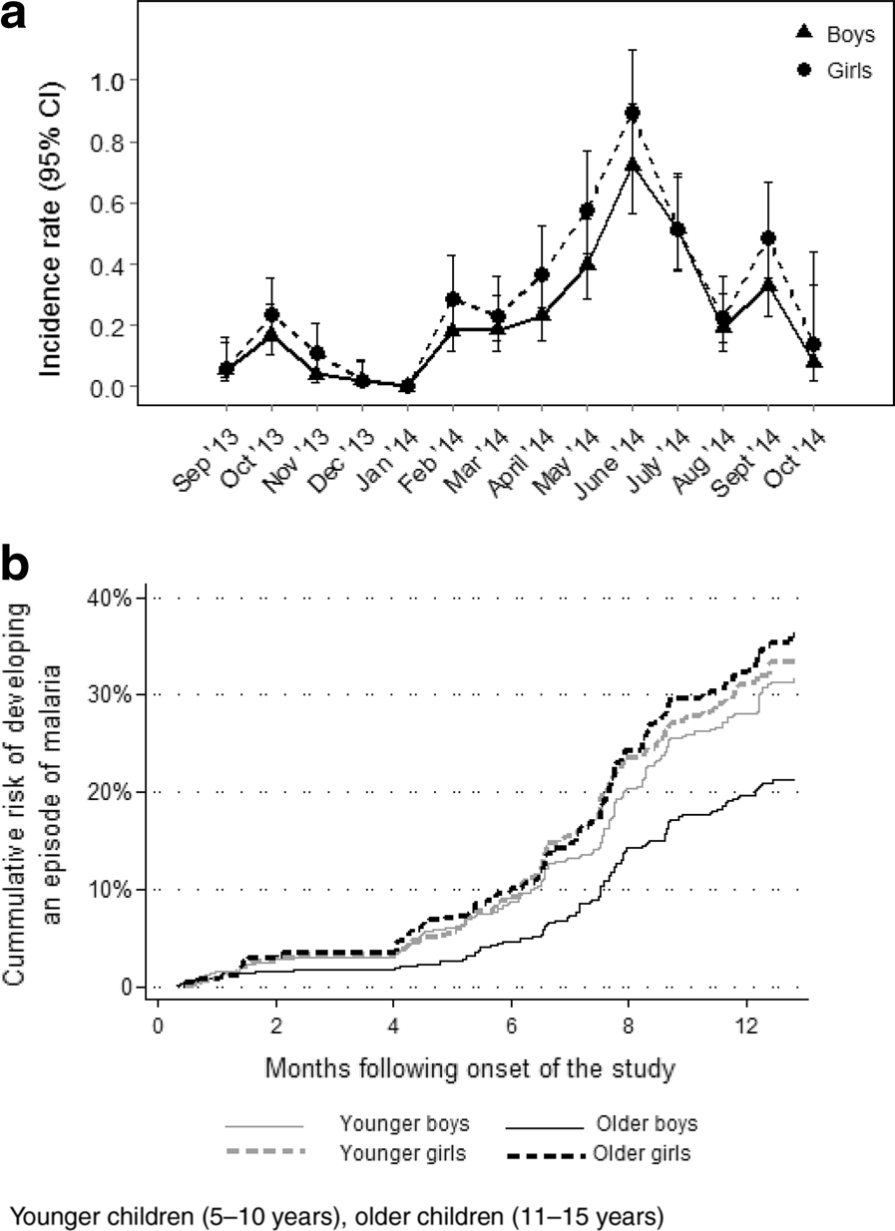
**a** Incidence rate of clinical malaria by sex and calendar month. **b** Cumulative risk of developing an episode of malaria by sex and age group over the 13 months follow-up among school children in Bumula District, western Kenya. Younger children (5–10 years), older children (11–15 years)

The total observation time during the 13 months of active case detection was 2310.8 person years, which was 85 % of the potential follow-up time. During the 13 months follow-up, 606 cases of malaria were recorded, corresponding to an overall incidence rate of 0.26 (95 % CI, 0.24–0.29) episodes per person-year. Of the 2346 children enrolled into the study, 1815 (77.4 %) did not experience any episode of clinical malaria during the follow-up period, 405 (17.3 %) children had only one episode and 93 (4 %) had two or more episodes of malaria. Having any episode and repeated episodes of clinical malaria was significantly higher among girls than boys (0.31 vs 0.23 episodes per person-year, P < 0.001) (Table 2; Fig. 2a) and girls were more likely to have repeated episodes of clinical malaria (P = 0.03). However, using alternative case definition with a parasite density cutoff of >2500 parasites/μL as used in previous studies [34], the incidence rate did not vary by sex (Table 2). Younger children (5–10 years) were 20 times more likely to have an episode of clinical malaria compared to the older age group (11–15 years) (P = 0.038). Although there were more girls in the younger age groups compared to boys, the incidence of malaria was higher in the older girls as shown in Fig. 2b with a higher cumulative risk of developing an episode of clinical malaria P < 0.001.

Incidence of malaria varied considerably by calendar month ranging from (0.00 to 0.80) episodes per person year with the peak corresponding with the wet season. When stratified by season and sex, the incidence rate varied among boys and girls between the seasons with broadly similar trends, (Table 2; Fig. 2a). In total, 361 (99 %) blood smears done during the wet season were confirmed as clinical malaria cases, compared to 248 (38 %) of the blood smears during the dry season.

In univariable analysis with season as a fixed term, clinical malaria was associated with being female (Incidence rate ratio 1.33, 95 % CI: 1.11–1.62), (Additional file 3). None of the other investigated factors were significant after adjusting for season and sex in multivariable analysis.

### Geographic distribution of *P. falciparum* infection and clinical malaria

**Fig. 3:**
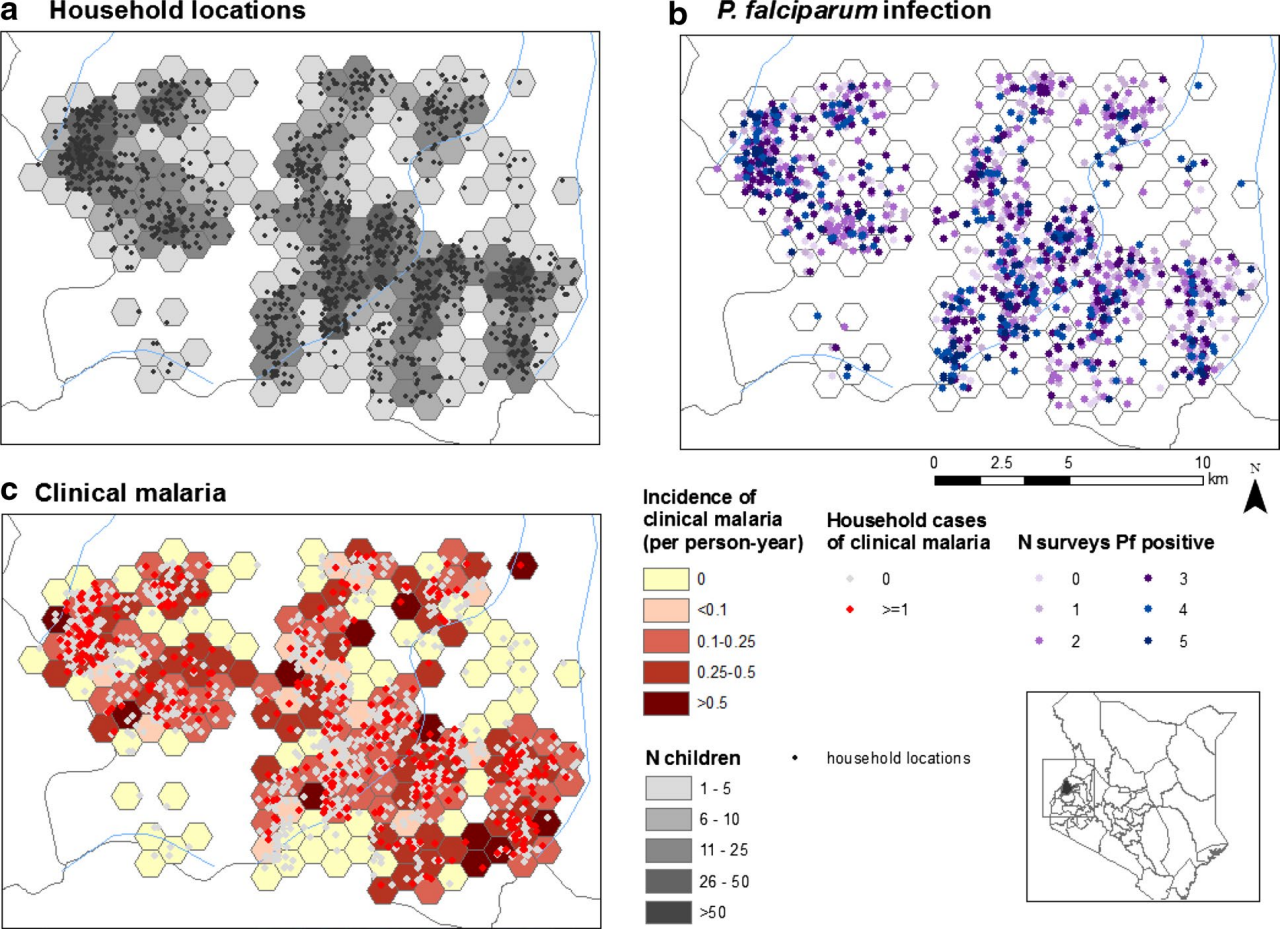
**a** Map of household locations, geographic distribution of **b** *Plasmodium falciparum* infection and **c** clinical malaria by household, during the 15 month follow-up period in Bumula district, western Kenya

**Fig. 4:**
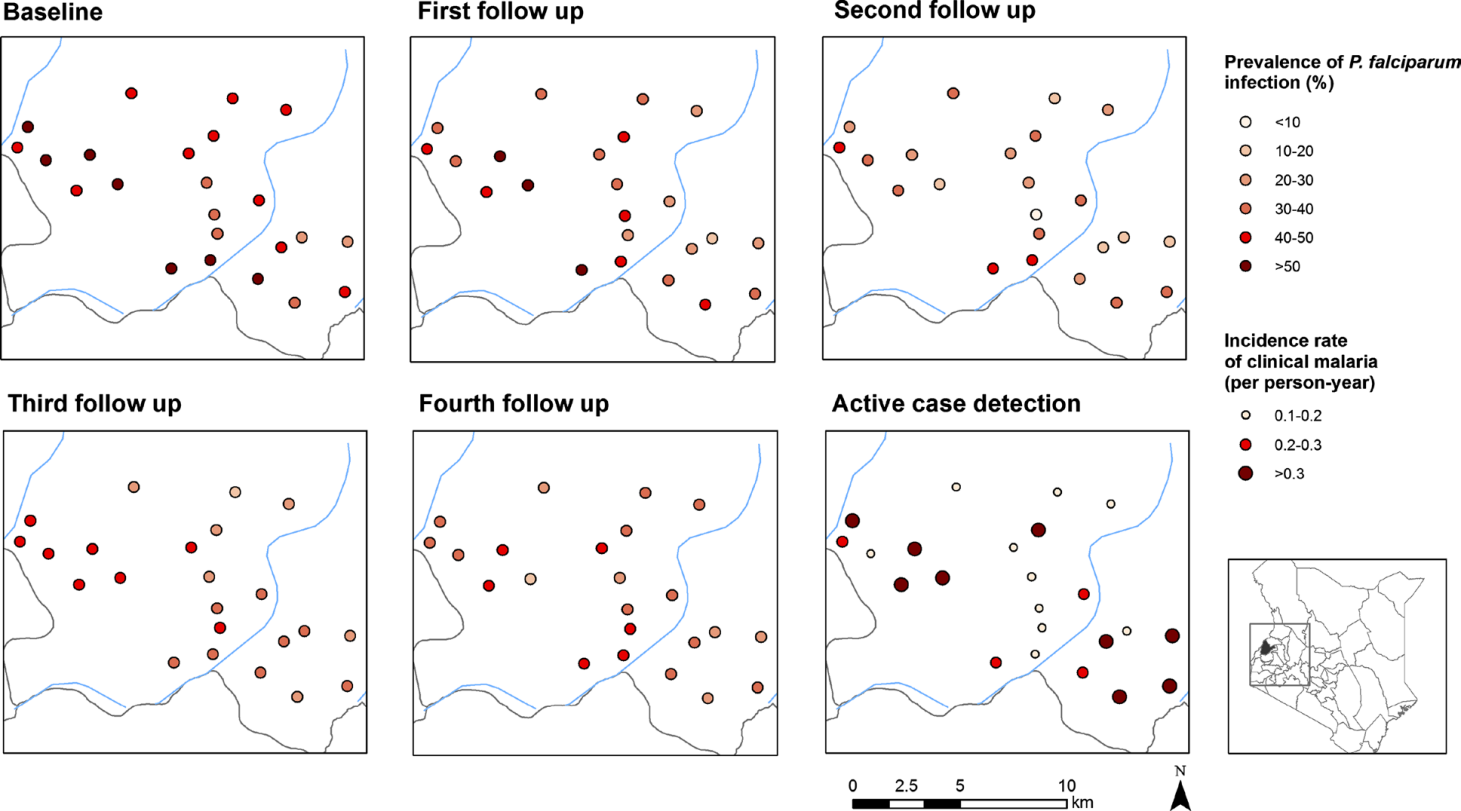
Geographic distribution of *Plasmodium falciparum* infection by survey and clinical malaria as identified by active case detection, by school

A map showing the location of households in the study area is provided in Fig. 3. Household GPS coordinates were missing for 408 children and sensitivity analysis showed that *P. falciparum* infection prevalence was higher among children with missing coordinates. Figure 3 shows the geographic distribution of clinical malaria and *P. falciparum* infection by households and survey round. *P. falciparum* infection occurred across the study area, but a subset of households in the west appeared to exhibit repeated *P. falciparum* infection with children from these households having 4–5 detected infections. Clustering of both *P. falciparum* infection and clinical malaria was more evident at school level, and followed a similar pattern to what was observed at household level (Fig. 4).

**Fig. 5:**
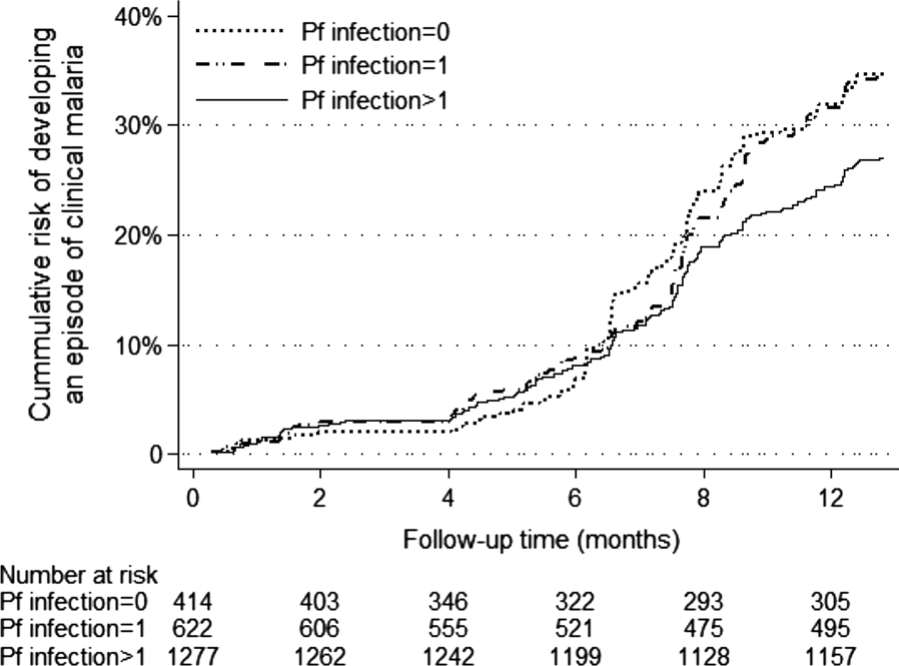
Cumulative risk of developing an episode of clinical malaria, over the 13 months follow, by *Plasmodium falciparum* infection during the cross-sectional survey among school children. Abbreviations: *Pf* *Plasmodium falciparum*; Pf = 0 no *P. falciparum* infection; Pf = 1 single *P. falciparum* infection; Pf = 2 repeated (more than once) *P. falciparum* infections during the five surveys conducted

Distribution of clinical malaria was skewed with majority of the children not experiencing any episode of clinical malaria (Fig. 4). Children with a single or no infection of *P. falciparum* during cross-sectional surveys had a higher cumulative risk for developing clinical malaria compared to children with repeated *P. falciparum* infection with a divergence of the survival curves occurring after 4 months as shown in Fig. 5 (P = 0.012). Only 11 % (364) of children did not experience any clinical malaria episode or *P. falciparum* infection.

### Anaemia

Overall, 834 children (38 %) were anaemic and anaemia was most common among 11–15 year-olds and among boys (Table 1). Among boys, anaemia was higher in the older age group (11–15 years) compared to 5–10 year-olds (P = 0.009), while in girls, although not significant, anaemia was higher among the younger age group (5–10 years) (P = 0.085). In univariable and multivariable analysis, anaemia was associated with being male, thinness, and presence of *P. falciparum* infection irrespective of number of times infected (Table 4).

**Table 4:** Factors associated with prevalence of anaemia, among school children in Bumula District

Variable	Crude odds ratio (95 % CI)	P value	Adjusted^a^ odds ratio (95 % CI)	P value
Sex				
Boys	1		1	
Girls	0.76 (0.64–0.90)	0.002	0.79 (0.66–0.95)	0.009
Age categories (years)				
5–10	1		1	
11–15	1.19 (1.00–1.42)	0.053	0.14 (0.95–1.37)	0.150
Stunted	0.90 (0.54–1.51)	0.704	1.13 (0.78–1.61)	0.521
Underweight	0.86 (0.67–1.10)	0.232	0.91 (0.53–1.54)	0.721
Thin	1.49 (1.12–1.99)	0.006	1.45 (1.26–1.67)	0.013
Any *P. falciparum* infection				
No	1		1	
Yes	1.41 (1.10–1.81)	0.006	1.39 (1.03–1.86)	0.017
Cumulative *P. falciparum* infection				
None	1		1	
Single infection	1.39 (1.05–1.83)	0.112	1.42 (1.23–1.63)	<0.001
Multiple infections	1.37 (1.06–1.77)		1.40 (1.23–1.60)	
Incidence of clinical malaria				
None	1		1	
Single episode	1.02 (0.84–1.24)	0.790	1.05 (0.86–1.28)	0.837
Multiple episodes	1.11 (0.79–1.56)		1.15 (0.82–1.63)	
Average bed net use				
Never	1		1	
Sometimes	0.96 (0.78–1.19)	0.828	0.99 (0.80–1.23)	0.736
Always	0.89 (0.70–1.13)		0.92 (0.72–1.17)	
Socioeconomic status				
Poor	1		1	
Not poor	0.97 (0.94–1.00)	0.089	0.97 (0.94–1.00)	0.096

^a^Multivariable analysis adjusted for Sex, being thin, and *P. falciparum* infection, P value base on likelihood ratio test, Any *P. falciparum* infection being infected in at least one of the surveys

## Discussion

The epidemiology of malaria among school children has previously received little attention, with few studies looking at factors associated with the risk among African school children [12, 35]. However there is a renewed interest because of the shift from malaria control that targets high risk groups to a more inclusive approach and targets community-wide transmission [36]. There is, therefore, need for robust data for all age-groups on the burden of malaria to inform planning of control programmes. These results show that malaria burden among school children was considerable, with at least 30 % of children being infected with *P. falciparum* at any one of five surveys and one in five children experiencing an attack of clinical malaria. The current study showed that asymptomatic malaria was common among boys, stunted and anaemic children from households of lower socioeconomic status, while the incidence of clinical malaria was associated with being female.

The sex difference in *P. falciparum* infection has previously been reported in Africa [37–40]. Although studies on sexual dimorphism have been inconclusive and warrant further investigation, hypotheses of underlying reasons for such sex differences include estrogen or testosterone specific modulation of antiplasmodial immune [41]. Moreover, the lower risk of clinical malaria among boys may be attributed to development of partial immunity as a result of repeated exposure [42]. This is further supported by the observed lower cumulative risk in children with repeated detections of *P. falciparum* infection, as it was boys who were more likely to have *P. falciparum* infection at any one of the surveys. Even though the reported sex differences are interesting from an epidemiological perspective, the programmatic significance of these findings are comparably low. *P. falciparum* infection prevalence was high among boys and girls and, therefore, both should be equally considered in malaria control strategies.

Furthermore, in this study anaemia was prevalent (two in five children), and was shown to be associated with *P. falciparum* infection. Similar findings have been consistently reported among school-aged populations in Africa [17, 22, 43–46]. The mechanism through which *Plasmodium* infection causes anaemia is multifactorial and includes direct destruction of infected red blood cells (RBCs), rupturing of RBCs, hypersplenism, and reduced RBC production in the bone marrow [47, 48]. Both anaemia and *P. falciparum* infection were associated with poor nutritional status: thinness and stunting, respectively. Malnourished children have previously been shown to have increased risk of malaria [46]. As micronutrient deficiency is common in many malaria endemic areas, it is plausible that these children may have been anaemic initially and *P. falciparum* infection further added to the strain of anaemia [49]. In addition, intestinal helminth infections are common in Bumula [50] and may also contribute to the observed prevalence of anaemia, although no association was observed between anaemia and hookworm infection in the baseline survey.

The observed association of *P. falciparum* with low socioeconomic status is in agreement with previous studies that show malaria is more common among people of lower socioeconomic status who often live in poorly constructed houses increasing their exposure to infection [4, 24, 30, 51]. Bumula is one of the poorest districts in Kenya, with 79 % of the households having their floors and walls made of mud and 40 % having a grass thatched roof [52]. In this study 95 % of the children came from houses with walls and floors made of mud and 10 % had thatched roofs. Such house constructions have been found associated with higher malaria prevalence and indoor vector density [53, 54] while improved house structure and quality of construction material was associated with lower incidence of malaria [55]. Modern housing is suggested to offer protection by obstructing mosquito vector entry and reducing their density in the houses, as compared to traditional houses that have thatched roofs, walls and floors made of mud [56, 57]. Improvement of socioeconomic development has been shown to be an effective intervention against malaria [58].

In contrast to cross sectional studies conducted in various parts of Africa, where bed net use has been shown to be lowest among school children [23], 89 % of the children reported sleeping under a bed net the previous night at the baseline survey. Moreover, bed net use was not associated with reduced risk of *P. falciparum* infection or clinical malaria as would be expected, however the finding is agreement with the last malaria indicator survey that did not show an association of bed net use and *Plasmodium* infection in Lake Victoria region [31]. It is possible, although children reported ownership of a bed net, they do not consistently use it, as only 22 % stated to consistently sleep under a bed net. Additionally, most school children in rural parts of Africa sleep on the floor and therefore hanging the net may be a challenge [24, 30]. An alternative explanation for the lack of protective effect of bed nets in this study may relate to development of insecticide resistance among *Anopheles* spp. mosquitoes as previously has been reported in western Kenya [59].

As expected, the prevalence parasitaemia and incidence of clinical malaria fluctuated depending on the rainfall patterns. Western Kenya is a high malaria transmission area with a annual precipitation ranging 1200–1800 mm [60]. Higher prevalence of infection and incidence of clinical malaria were observed during the wet season, consistent, with what is known, transmission intensity varies with rainfall patterns [61]. Despite this seasonal variation, observed *P. falciparum* infection prevalence was important also during the dry season suggesting that transmission control interventions should not only be targeted to the wet season. Mapping of *P. falciparum* infection and clinical malaria by survey at school and household level showed pronounced spatial clustering in the western part of the study area. This may be explained by an insufficient spatial resolution of the environmental data used according to the size of the study area. It is well known that the major malaria vectors in the area (*Anopheles gambiae* s.s.) has greater affinity for small breeding sites such as animal footprints and small ponds formed during rainy season [62] which is hardly detected by medium resolution satellite imagery, such as those used in this study. Clustering was more evident at school level compared to households, suggesting that school based surveys may provide valuable insight into community transmission dynamics. School based surveys have been previously shown to provide a more cost-effective framework for the planning and evaluation of malaria control programmes [10]. It is more effective to survey children at schools compared to community based surveillance which often misses them [13].

The major strength of this study was the temporal dimension afforded by examination of a cohort over 1½ years, with 85 % follow-up rate. The study had a number of limitations. First, the children included in the main trial were from schools that were purposively selected based on accessibility, and therefore may not necessarily be representative of all school-aged children in the study area. However, these findings are similar to a study based on a representative sample of schools conducted in western Kenya [63]. Second, diagnosis was based on routine parasitological procedures and expert malaria microscopy may miss light infections when compared to more sensitive molecular methods [28]. School children have been shown in previous studies, to contribute substantially to sub microscopic infection and therefore the true prevalence may have been under estimated [13, 28]. Third, clinical malaria episodes were missed out if treatment was sought outside the study. Fourth, the age or quality of the bed net and relied on self-reported use. Bed nets may have been of poor condition or torn and therefore fail to provide adequate protection as has been previously reported in Kenya [64–66]. Fifth, haemoglobin level was only measured at baseline; a better assessment of the association of anaemia and clinical malaria could have been made by measuring haemoglobin levels for each suspected case.

## Conclusion

The results show that the burden of clinical malaria, *P. falciparum* infection and anaemia was high among school children in Bumula district, western Kenya. Although reported bed net ownership was high, consistent use was low. Therefore, promoting bed net use among this age group may help to achieve the desired protective effect of this intervention. The study demonstrated the need to include school children in standard malaria interventions, which may alleviate the observed high anaemia burden.

## Additional files

10.1186/s12936-016-1176-y Baseline characteristics among school children who were infected with any STH versus those who were uninfected in Bumula district. The table compares baseline characteristics of study children who were infected with any soil transmitted helminth versus those who were uninfected at recruitment.
10.1186/s12936-016-1176-y Sources and processing of environmental data. A summary of sources and processing procedures of environmental data is provided.
10.1186/s12936-016-1176-y Factors associated with incidence of clinical malaria among school children in Bumula, district. This table shows analysis of risk factors associated with clinical malaria.
