# Supplementary material for: Plasmodium falciparum parasitaemia and clinical malaria among school children living in a high transmission setting in western Kenya
Source: Malar J. 2016 Mar 11;15:157. doi: 10.1186/s12936-016-1176-y (PMC4788950; doi:10.1186/s12936-016-1176-y)
Supplement: Supplementary file 1 — 10.1186/s12936-016-1176-y Baseline characteristics among school children who were infected with any STH versus those who were uninfected in Bumula district. The table compares baseline characteristics of study children who were infected with any soil transmitted helminth versus those who were uninfected at recruitment. [file 12936_2016_1176_MOESM1_ESM.doc]

**Additional file 1**

**Supplementary Table 1: Baseline characteristics among school children who were infected with any STH versus those who were uninfected in Bumula district.**

| **Characteristic** | **Uninfected**  **N=841 (%)** | **Infected with Any STH**  **N=1,505 (%)** | **^1^P value** |
| --- | --- | --- | --- |
| *Child Characteristics* |  |  |  |
| Sex, girls | 422 (50.2) | 692 (46.0) | 0.051 |
| Age, years, mean (SD) | 10.4 (2.5) | 10.5 (2.4) | 0.494 |
| Age categories (years) |  |  |  |
| 5-10 | 414 (49.2) | 764 (50.8) |  |
| 11-15 | 427 (50.8) | 741 (49.2) | 0.475 |
| Mean body temperature, mean ^o^C (SD) | 36.4 (0.02) | 36.6 (0.02) | 0.616 |
| WAZ <-2 SD below median reference value | 58 (14.0) | 116 (15.2) | 0.588 |
| HAZ <-2 SD below median reference value | 44 (3.6) | 27 (2.4) | 0.891 |
| BMIZ <-2 SD below median reference value | 26 (3.1) | 45 (3.0) | 0.001 |
| Malaria parasitaemia | 607 (50.8) | 488 (46.7) | 0.015 |
| Parasitaemia, parasites/μL, mean (95% CI) | 2008 (1231-2786) | 1818 (1247-2390) | 0.699 |
| Hemoglobin, g/dL, mean (SD) | 12.3 (0.05) | 12.3 (0.04) | 0.446 |
| Anaemia | 289 (36.7) | 545 (38.7) | 0.347 |
| *Household characteristics* |  |  |  |
| Slept under a bed net previous night | 322 (68.4) | 958 (63.7) | 0.061 |
| Education level of household head |  |  |  |
| None or incomplete primary | 430 (56.1) | 825 (58.2) | 0.339 |
| Above primary school | 337 (43.9) | 593 (41.8) |  |

Abbreviations: SD, standard deviation; WAZ, weight-for-age z-score (underweight); HAZ, height-for-age z-score (stunted); BMIZ, body mass (thin); CI, confidence interval; μL, microlitre ; g/dL ; grammes/decilitre

^1^Based on wald test
