# Supplementary material for: Plasmodium falciparum parasitaemia and clinical malaria among school children living in a high transmission setting in western Kenya
Source: Malar J. 2016 Mar 11;15:157. doi: 10.1186/s12936-016-1176-y (PMC4788950; doi:10.1186/s12936-016-1176-y)
Supplement: Supplementary file 2 — 10.1186/s12936-016-1176-y Sources and processing of environmental data. A summary of sources and processing procedures of environmental data is provided. [file 12936_2016_1176_MOESM2_ESM.doc]

**Additional file 2**

*Sources and processing of environmental data*

Elevation data at 250m resolution were derived from gridded digital elevation models (DEM) produced by the Shuttle Radar Topography Mission (SRTM). Averaged long-term enhanced vegetation index (EVI) for the period 2000 to 2013 was obtained from the Africa Soil Information Service (AfSIS) [1]. This vegetation index is collected at a 16-day basis by the *Moderate Resolution Imaging Spectroradiometer* (MODIS) sensor and delivered in monthly average raster datasets at 250 m resolution by the Columbia University International Research Institute for Climate and Society (IRI).

Land surface temperature (LST) and normalized difference vegetation index (NDVI) were estimated from a suite of remotely sensed imagery of the study area obtained through the Landsat-8 Operational Land Imager (OLI) sensor at different period of times (Figure 1S). A level-1 product was downloaded from the U.S. Geological Survey (USGS) website [2] (Figure S2). In order to increase the spatial resolution of multispectral bands (provided at 30m resolution) a pan-sharpening process was applied, except for thermal infrared bands, using the 15-m panchromatic band. Atmospheric correction was then conducted for the bands 1 to 8 using the DOS model-based algorithm and assuming 1% minimum reflectance [3]. Radiance and ground reflectance were subsequently obtained for each band [4]. The thermal infrared band (TIR) 10 (10.60 - 11.19 µm) was used to estimate the effective at-sensor brightness temperature (
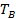
) using the Plank’s inverse functions as detailed elsewhere [5]. The final LST was estimated by the following equation:


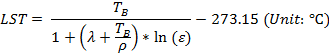


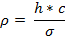


where,
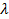
 is the wavelength of the emitted radiance which is equal to 10.8 µm;
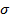
 is the Stefan Boltzmann’s constant which is equal to 5.67 x 10^-8^ Wm^-2^ K^-4^ ;
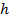
 is the Plank’s constant (6.626x10^-34^ JSec);
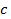
is the velocity of light (2.998x10^8^ m/sec) and
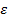
 is the spectral emissivity which was estimated as follows:


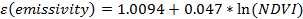


The spectral bands 4 (red: 0.64 - 0.67 µm) and 5 (NIR: 0.85 - 0.88 µm) were used to generate NDVI raster dataset of the study area (Figure S1), whilst for the normalized difference water index we used a modified version suggested by Xu (2006) [6, 7] which is based on a combination of the bands 3 (green: 0.53 - 0.59 µm) and 6 (SWIR1: 1.57 - 1.65 µm).

NDVI = NIR-RED/NIR+RED

MNWDI: GREEN-SWIR1/ GREEN+SWIR1

We cross-validated our MNWI raster dataset using a suite of field points collected from high-resolution satellite images obtained from Digital Globe for the period of study. We created a list of 150 random field points which fell in water areas according to the high resolution images. For this set of points we extracted the corresponding MNWI value and fitted a ROC curve. The cutoff value which optimized the sensitivity and specificity in the ROC curve was used to discriminate water bodies for each image. Seasonal and permanent water bodies were identified through combining the different binary gridded maps obtained for each time. Thus, water areas depicted in dry and rainy season maps were considered permanent water bodies and seasonal water bodies when were present in only one of the seasons. Finally, Euclidean distance (straight-line distance) from the schools to the nearest water body, both permanent and seasonal water bodies, was calculated.

**References**

| **Figure S1** Period of times for the acquisition of satellite imagery captured through the Landsat-8 Operational Land Imager (OLI) sensor between 2013 and 2014  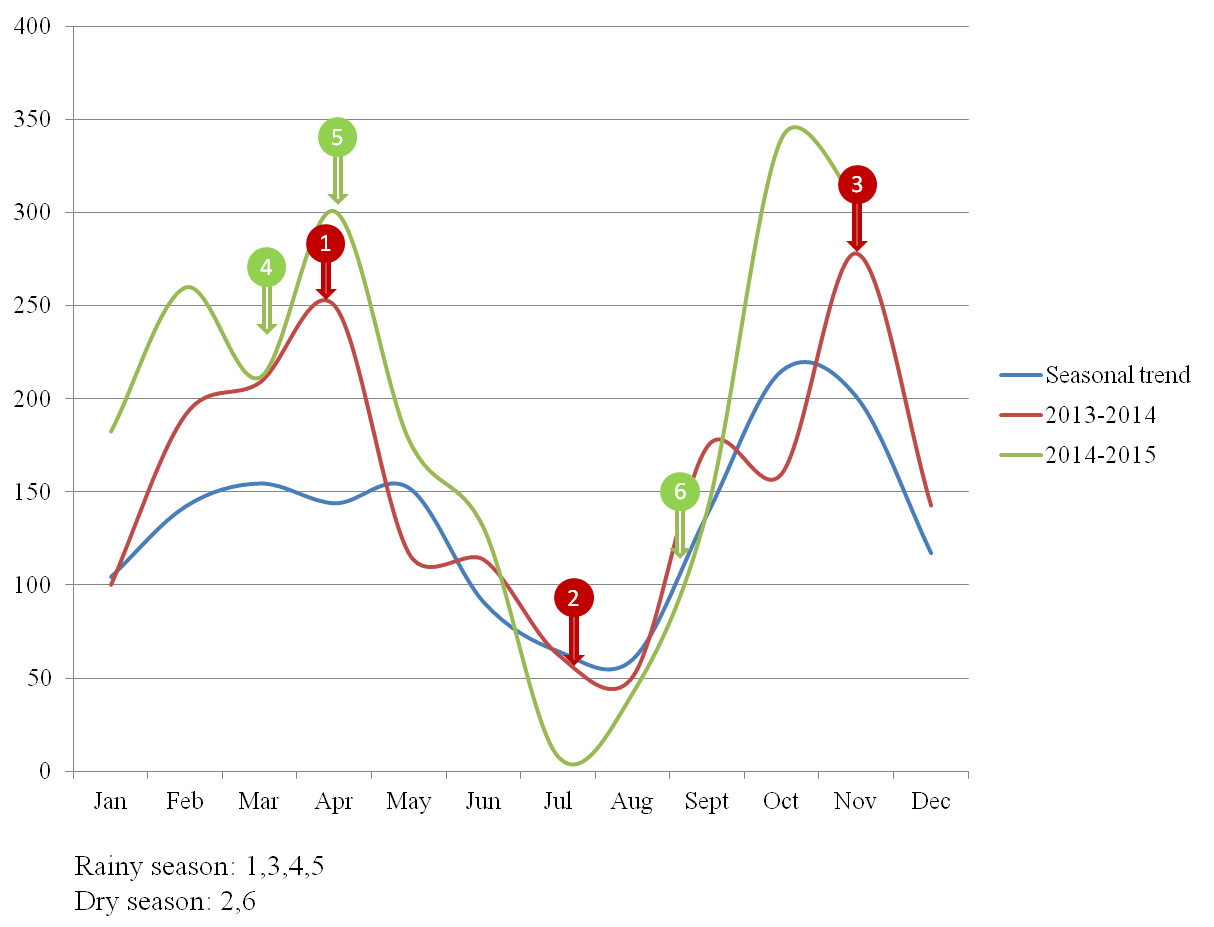  **Figure S2** Land surface Temperature, NDVI, NDWI and water bodies obtained from satellite imagery (Landsat-8 OLI) of the study area at September, 2014. |
| --- |
| **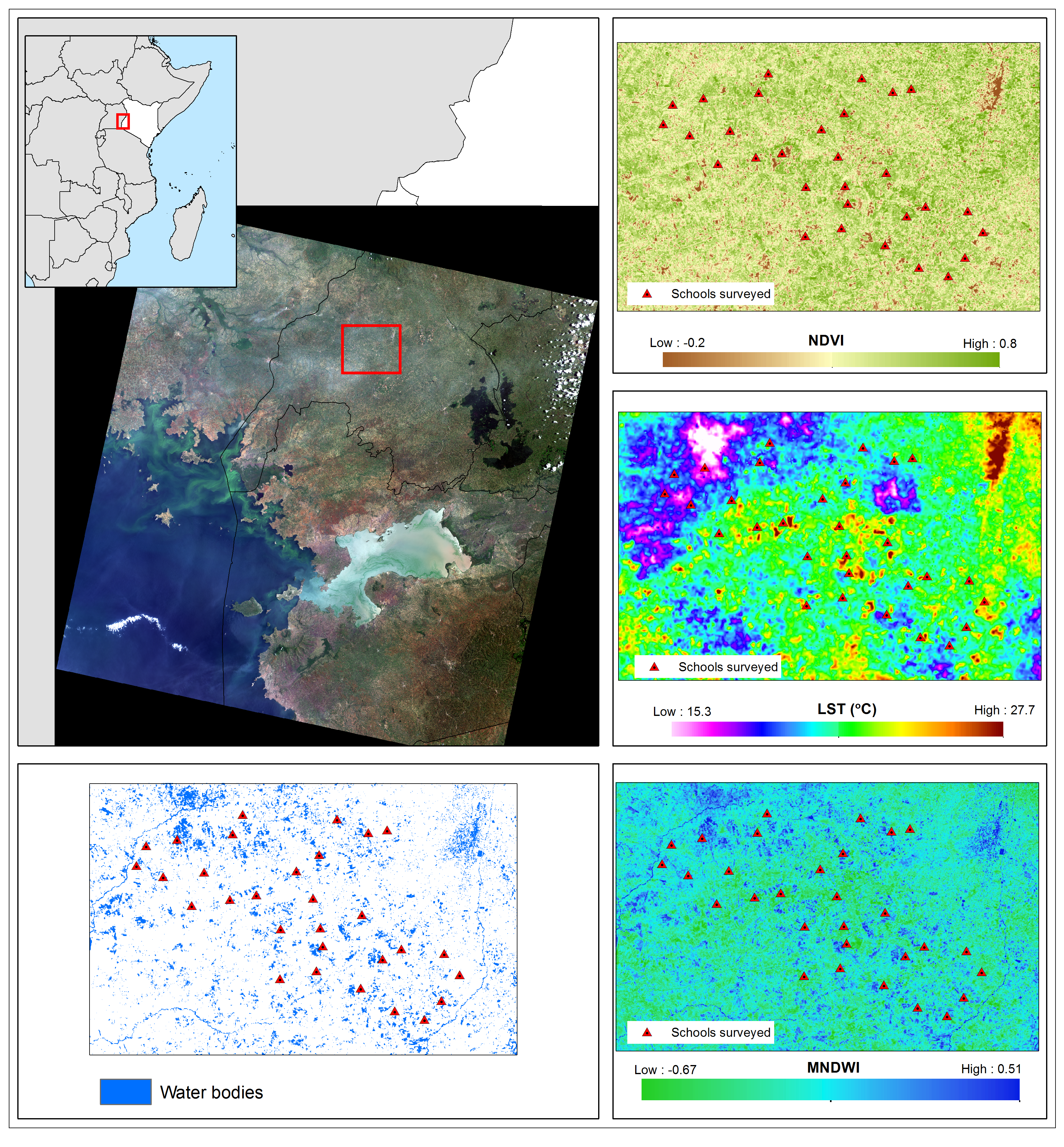** |
