# Supplementary material for: Plasmodium falciparum parasitaemia and clinical malaria among school children living in a high transmission setting in western Kenya
Source: Malar J. 2016 Mar 11;15:157. doi: 10.1186/s12936-016-1176-y (PMC4788950; doi:10.1186/s12936-016-1176-y)
Supplement: Supplementary file 3 — 10.1186/s12936-016-1176-y Factors associated with incidence of clinical malaria among school children in Bumula, district. This table shows analysis of risk factors associated with clinical malaria. [file 12936_2016_1176_MOESM3_ESM.doc]

**Additional file 3**

**Supplementary Table 2: Factors associated with incidence of clinical malaria among school children in Bumula, district.**

| **Variable** | **Episodes** | **Persons years** | **Incidence per person year** | **Univariable analysis** | |
| --- | --- | --- | --- | --- | --- |
|  |  |  |  | **IRR (95% CI)** | **P value** |
| *Individual characteristics* |  |  |  |  |  |
| Sex |  |  |  |  |  |
| Boys | 277 | 1218.9 | 0.23 | 1 |  |
| Girls | 329 | 1091.9 | 0.30 | 1.33 (1.11-1.62) | 0.003 |
| Age categories (years) |  |  |  |  |  |
| 5-10 | 329 | 1153.9 | 0.28 | 1 |  |
| 11-15 | 277 | 1156.9 | 0.24 | 0.89 (0.74-1.08) | 0.252 |
| Not stunted | 460 | 1686.3 | 0.27 | 1 |  |
| Stunted | 146 | 582.2 | 0.25 | 0.93 (0.74-1.15) | 0.504 |
| Not under weight | 586 | 2195.9 | 0.24 | 1 |  |
| Under weight | 20 | 72.6 | 0.17 | 1.18 (0.70-2.00) | 0.527 |
| Not thin | 545 | 2025.6 | 0.27 | 1 |  |
| Thin | 61 | 242.9 | 0.25 | 0.91 (0.66-1.25) | 0.566 |
| Not Anaemic | 356 | 1354.3 | 0.26 | 1 |  |
| Anaemic | 237 | 818.8 | 0.29 | 1.06 (0.86-1.29) | 0.593 |
| *Household characteristics* |  |  |  |  |  |
| Bed net use |  |  |  |  |  |
| No | 453 | 1704.1 | 0.27 | 1 |  |
| Yes | 122 | 453.6 | 0.27 | 0.92 (0.73-1.16) | 0.508 |
| Usually sleeps under a bed net |  |  |  |  |  |
| Never | 285 | 874.2 | 0.33 | 1 |  |
| Sometimes | 195 | 760.4 | 0.26 | 0.83 (0.67-1.04) | 0.703 |
| Always | 116 | 554.2 | 0.21 | 0.64 (0.50-0.84) |  |
| Socio economic status |  |  |  |  |  |
| Poor | 351 | 1351.9 | 0.26 | 1 |  |
| Not poor | 215 | 789.8 | 0.27 | 1.00 (0.98-1.03) | 0.856 |

Stunted =HAZ <-2 SD below median reference value, thin=BMIZ <-2 SD below median reference value, underweight=WAZ <-2 SD above median reference value
